# Toward Light‐Regulated Living Biomaterials

**DOI:** 10.1002/advs.201800383

**Published:** 2018-06-29

**Authors:** Shrikrishnan Sankaran, Shifang Zhao, Christina Muth, Julieta Paez, Aránzazu del Campo

**Affiliations:** ^1^ INM – Leibniz Institute for New Materials Campus D2 2 66123 Saarbrücken Germany; ^2^ Chemistry Department Saarland University 66123 Saarbrücken Germany

**Keywords:** dynamic biomaterials, endotoxin‐free *E. coli*, living biointerfaces, optogenetics

## Abstract

Living materials are an emergent material class, infused with the productive, adaptive, and regenerative properties of living organisms. Property regulation in living materials requires encoding responsive units in the living components to allow external manipulation of their function. Here, an optoregulated *Escherichia coli (E. coli)*‐based living biomaterial that can be externally addressed using light to interact with mammalian cells is demonstrated. This is achieved by using a photoactivatable inducer of gene expression and bacterial surface display technology to present an integrin‐specific miniprotein on the outer membrane of an endotoxin‐free *E. coli* strain. Hydrogel surfaces functionalized with the bacteria can expose cell adhesive molecules upon in situ light‐activation, and trigger cell adhesion. Surface immobilized bacteria are able to deliver a fluorescent protein to the mammalian cells with which they are interacting, indicating the potential of such a bacterial material to deliver molecules to cells in a targeted manner.

The development of biomaterials able to provide signals to living cells and support basic cellular functions is an active area of research with potential for industrial and medical translation.^1^ Research efforts nowadays are particularly dedicated toward creating dynamic biomaterials enabling active regulation of signaling molecules (adhesive ligands or growth factors) or matrix mechanics upon application of external stimuli.^2^ Light has emerged as an advantageous stimulus in this context. It allows non‐invasive, spatiotemporally resolved activation, tunable regulation by varying exposure dose, and multiplexing by using different wavelengths. Light‐regulated ligand availability,^3^, ^4^ material stiffness,^5^, ^6^ topography,^7^, ^8^ and degradation^6^, ^9^ have been achieved in biomaterials through the introduction of synthetic chromophores either in the material or the ligand structure. However, most of these approaches are irreversible, cannot be reactivated, and require specific chemical design and extensive synthesis for each individual ligand or polymeric chain.^2^ We do not have strategies to remodel biomaterials in a truly dynamic manner and involving complex molecules, as it happens in living tissues.^10^ The first steps to circumvent some of these limitations is presented in this work. It is based on the incorporation of a photoregulated living component, such as optogenetically engineered bacteria, to a biomaterial (hydrogel) to form a light‐regulated living biomaterial. Upon light exposure, the bacteria are induced to produce proteins of interest in situ, and actively remodel the composition of the microenvironment. This article describes the biomaterial design and demonstration of the working principle using bacteria producing a simple cell‐adhesive miniprotein to tune cellular attachment to the living hybrid with light (**Figure** **1**a). While reversibility and spatial confinement of light‐responsive gene regulation could not be easily demonstrated in the current design, the flexibility of this approach allows easy reengineering with alternative optogenetic strategies and all kind of relevant biological factors to guide cellular processes in the future.

**Figure 1:**
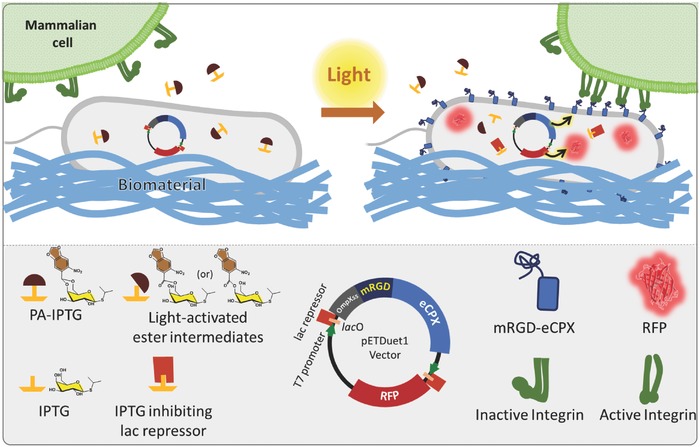
Molecular design of the light‐inducible *E. coli‐*biomaterial system. PA‐IPTG used along with an IPTG‐inducible dual expression vector enables light‐controlled protein expression. Upon light irradiation, PA‐IPTG gets converts to an ester intermediate that is enzymatically hydrolyzed into active IPTG within *E. coli*. The free IPTG inhibits the lac repressor and starts production of the genetically encoded proteins, mRGD‐eCPX and RFP. The transmembrane carrier protein eCPX allows surface display of the mRGD at the bacterial membrane. RFP acts as a reporter to identify the activated bacterial cells. Integrins at the surface of mammalian cells interact with mRGD displayed on the bacterial surface and mediate cellular attachment to the bacterial biomaterial.

Salmeron‐Sanchez and co‐workers have pioneered the concept of bacterial biointerfaces, based on *Lactococcus lactis*.^11^, ^12^, ^13^ Poly(ethyl acrylate) substrates coated with *L. lactis* displayed a fibronectin fragment on their outer surfaces and were used to guide stem cell differentiation. This fascinating approach opened a new window for dynamic biomaterials design. However, inducible protein expression in *L. lactis* is relatively complex and there are no available light‐induced gene expression strategies in this system. Being the workhorse of genetic engineering, innumerable systems have successfully engineered *Escherichia coli (E. coli)* for bacterial ligand display,^14^, ^15^, ^16^ secretory protein expression,^17^, ^18^, ^19^ metabolic engineering,^20^, ^21^, ^22^ non‐natural amino acid incorporation,^23^ etc. Optogenetic strategies to regulate these processes by light are also available.^24^, ^25^, ^26^, ^27^ Here we demonstrate the use of bacterial surface‐display in combination with optogenetics in *E. coli* to dynamically regulate the interaction of living materials with mammalian cells using light. The commonly used *E. coli* lab strains neither form biofilms nor are known to secrete endogenous proteins. They can grow rapidly but do not form spores or dormant morphologies and they can be eliminated using common antibiotics. For these reasons, *E. coli* is an ideal living component in biomaterials to be engineered to provide multiple intricate functions.

A commercially available genetically engineered endotoxin‐free strain named ClearColi BL21(DE3), in which gene expression can be activated by the widely used inducer, isopropyl β‐d‐1‐thiogalactopyranoside (IPTG), was used in this study.^28^ In order to introduce light response, a photoactivatable version of IPTG (PA‐IPTG) was synthesized.^24^, ^25^ The *E. coli* strain was engineered to display an RGD‐containing miniprotein (mRGD)^29^ on its surface using an enhanced circularly‐permutated outer membrane protein X (eCPX).^14^, ^15^ This mRGD‐eCPX fusion protein was introduced in the bicistrionic vector, pET‐Duet1, along with the red fluorescent protein, TagRFP (RFP), as a reporter to visualize activation of protein expression in the bacterial cells (Figure 1). The expression of both genes is simultaneously induced in the presence of IPTG. A negative control strain was also engineered in which mRGD was replaced by the scrambled version, mRDG.^29^ The bacterial strain expressing mRGD‐eCPX will be referred to as *E. coli*+, and the one expressing the scrambled mRDG‐eCPX as *E. coli*−. To verify whether mRGD was being displayed on the bacterial surface, the cells were lysed and fractionated to isolate the outer membrane from the rest of the cell. Western blotting allowed us to clearly identify the presence of mRGD‐eCPX‐His6 only in the outermembrane fraction (**Figure** **2**a). The TagRFP construct also carried a His6 tag and was visualized in both fractions, in agreement with a recent finding that shows auto‐secretion of fluorescent proteins overexpressed in *E. coli*.^19^

**Figure 2:**
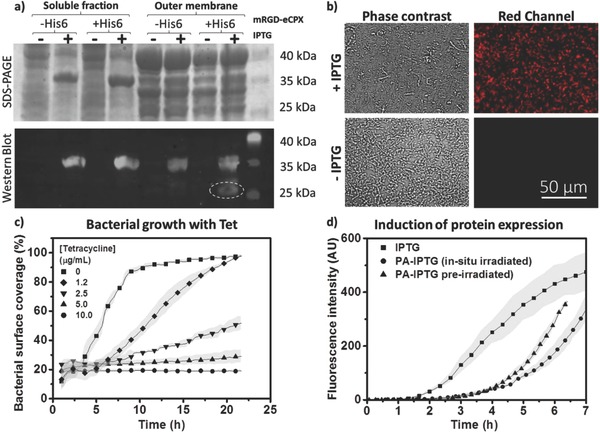
a) SDS‐PAGE and western blot analyses performed on bacterial lysates fractionated into soluble and outermembrane fractions. RFP carries an N‐terminal His6 tag, while mRGD‐eCPX was His6‐tagged on its C‐terminal. Anti‐His6 primary antibody was used for western blot analysis and protein detection. The dashed circle represents His6‐eCPX‐mRGD. b) RFP expression of *E. coli +* cells immobilized on PDL‐coated surfaces. Expression was induced with IPTG and images were made 2 h after induction. In the absence of IPTG, red fluorescence is not observed in the bacteria. c) Surface coverage of bacterial cells in the presence of increasing tetracycline concentrations over time. Beyond 90% surface coverage, multilayers started to form but their bacterial content was not quantifiable in the current microscopy‐based assay and is represented as a saturation phase. d) Expression of RFP in surface‐immobilized *E. coli+* bacterial cells induced using either IPTG or PA‐IPTG that was pre‐irradiated or in situ irradiated with 360 nm light for 2 min. The gray bands in all plots represent standard deviation obtained from three individual samples in each case.

Poly‐d‐lysine (PDL) coated Nexterion slides were used for immobilizing bacteria and construct the light‐regulated living biointerface. *E. coli* surface is negatively charged due to its outermembrane phospholipids and lipopolysaccharides, and the bacteria adhered within a few minutes to the positively charged PDL surface with high surface coverage. Attached bacteria induced with IPTG were able to express the proteins on their surface, as indicated by the red fluorescence in the microscopy images (Figure 2b). Initially, we observed rapid bacterial growth on the surface, resulting in the formation of multilayers within 6 h. Retardation of bacterial metabolism was achieved by addition of tetracycline antibiotic.^12^ Bacterial growth and protein expression were followed over the span of a day in the presence of tetracycline at concentrations spanning 1–10 µg mL^−1^ (Figure S1a, Supporting Information). Increasing concentrations of tetracycline gradually slowed bacterial growth and completely arrested it at 10 µg mL^−1^ (Figure 2c). After removal of the antibiotic, protein expression occurred at a normal rate (Figure S1c, Supporting Information), indicating that the antibiotic did not leave any permanent damage to the bacterial protein expression machinery. It was also observed that tetracycline reduced protein expression, while the presence of IPTG partially retarded bacterial growth. Hence in further experiments, 10 µg mL^−1^ tetracycline was used before induction of gene expression and either 2 or 0 µg mL^−1^ was used after induction when the duration of the experiment was longer or shorter than 10 h, respectively. It is important to note that growth arrest switches can also be incorporated to the bacterial genome to avoid the use of antibiotics and develop more advanced versions of this approach.^30^

The ability of the surface‐immobilized bacteria to express the protein upon addition of the photoactivatable inducer PA‐IPTG and light exposure was tested. PA‐IPTG was added to the medium and, based on previously reported analysis of photoactivation of PA‐IPTG in the presence of bacteria,^25^ the bacterial biointerface was exposed for 2 min at 360 nm light using the microscope's DAPI‐channel LED light source. This extent of light exposure has been shown to elicit maximum protein expression in *E. coli*.^25^ Red fluorescence corresponding to expression of RFP, indicating simultaneous expression of mRGD‐eCPX was observed 3 h after exposure, indicating that *E. coli*+ retained metabolic activity and protein expression capability after the light exposure step (Figure 2d). Control experiments with IPTG and preirradiated PA‐IPTG solutions at equivalent concentrations were also performed. Whereas red fluorescence was observed 1.5 h after induction with soluble IPTG, in agreement with RFPs maturation half time of 100 min,^31^ the irradiated PA‐IPTG samples took ≈1.5 h longer to display visible levels of red fluorescence (**Figure** **3**a). The time between induction with PA‐IPTG and protein expression is intrinsic to this system and has been associated to the formation of photolysis intermediates that get hydrolyzed to active IPTG by machinery within the *E. coli* (Figure 1; Figure S2, Supporting Information).^24^, ^25^

**Figure 3:**
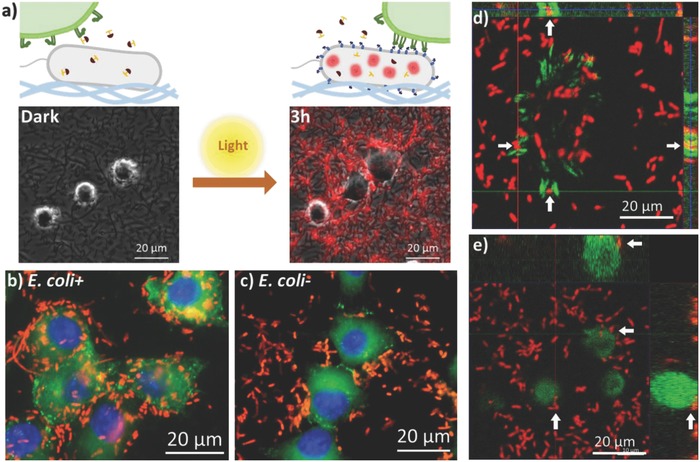
Optoregulated interactions between cells and bacterial material. a) Microscopy images (merged phase contrast and red fluorescence channels) of *E. coli +* surfaces seeded with MEF‐vincGFP cells in the presence of PA‐IPTG in the dark (left) and 3 h (right) after light exposure. b,c) Epifluorescence images of MEF‐vincGFP cells on *E. coli+* (b) and *E. coli*− (c) surfaces at 7 h after light activation. Images were taken after fixation. d,e) Laser scanning confocal *Z*‐stack orthogonal projection images of focal adhesions wrapped around *E. coli+* (d) and not around *E. coli*− (e). Images correspond to fixed samples 10 h after IPTG induction. Red: bacteria, Green: vincGFP in MEF‐vincGFP cells, Blue: DAPI. The white arrows indicate particular bacterial cells in both the XY plane image and the XZ and YZ orthogonal projections on the top and right, respectively. Arrows pointing in the same direction correspond to the same bacterial cell.

Once control of bacterial growth and light‐driven protein expression on the living biointerface were demonstrated, we tested the possibility to light‐control mammalian cell response on the light‐regulated biointerface. Mouse embryonic fibroblasts expressing vinculin‐GFP (MEF‐vincGFP) were used for these experiments in order to visualize the formation of focal adhesions in response to the light‐induced display of mRGD adhesive miniprotein on the surface of the bacteria. After addition of PA‐IPTG and cell seeding, the substrate was irradiated at one corner in order to prevent photodamage on the MEFs. Initially, MEF‐vincGFP cells did not show interaction with the bacterial biomaterial. Cells remained predominantly round and migrated very little (Figure 3a). Approximately 3 h after irradiation, bacteria started exhibiting red fluorescence. Shortly before that, the MEF‐vincGFP cells started to extend protrusions and pull on the bacteria around them (Video S1, Supporting Information). This observation indicates that the mRGD displayed at the bacterial surface was recognized by integrins at MEFs membrane even before fluorescence was detected by the microscope. This was most likely due to the different rates of expression of mRGD‐eCPX and RFP and due to RFPs maturation time.^31^ As time progressed, MEF‐vincGFP cells migrated across the substrate and bacteria were accumulated around and under them (Figure 3a,b). On surfaces with light‐activated *E. coli*−, the mammalian cells were also seen to migrate slowly, but they did not accumulate any bacteria. The accumulation of bacteria is a consequence of the traction forces applied by cells at the adhesion points, which seem to be larger than the adhesion force of the bacteria with the underlying substrate. Note that previous studies had shown that *E. coli* can strongly adhere to poly‐l‐lysine surfaces with rupture forces around 5 nN.^32^ Similar results were observed when the bacterial surfaces were chemically induced by adding IPTG to the medium (Figure S3, Supporting Information). MEF‐vincGFP cells seeded on control surfaces coated with *E. coli*− did not interact with the bacteria (Figure 3c). *Z*‐stack confocal imaging revealed that focal adhesions formed around *E. coli+* bacterial cells (Figure 3d), and not around *E. coli*− (Figure 3e). Scanning electron microscopy (SEM) imaging of MEF‐vincGFP with *E. coli+* revealed close contact between the mammalian and the bacterial cells, with cell protrusions completely covering the bacteria (Figure S4, Supporting Information). Despite the fact that *E. coli* has a complex outer surface with flagella, pili, carbohydrates, etc. the mammalian cells only interacted with them when they displayed mRGD, suggesting that *E. coli* provides an interface for establishing highly specific cellular interactions. These results demonstrate the possibility to in situ regulate cell–materials interactions using light exposure and optogenetically engineered bacteria as active components of the artificial microenvironment.

After 3 h of interaction between MEF‐vincGFP cells and induced *E. coli+* bacteria, faint red fluorescence was also observed in the intracellular space (Figure S5a, Supporting Information). Such red fluorescence was not observed in cells on the *E. coli*− bacterial surface, even after 20 h (Figure S5b, Supporting Information). No indication of bacterial lysis or endocytosis was observed by phase contrast, epifluorescence and SEM imaging of the surface. Based on the recent discovery that *E. coli* can secrete negatively charged β‐barrel‐shaped fluorescent proteins,^19^ we hypothesized that the RFP used as a protein‐expression indicator was secreted from the bacteria. To test this theory, *E. coli+* was grown and induced using IPTG at a high density in a shaking culture. SDS‐PAGE analysis of the culture medium after 16 h of protein expression revealed the presence of RFP (**Figure** **4**a). When Ni‐NTA coated agarose beads were incubated with small extracts of the culture media, the agarose beads became fluorescent already 3 h after induction, indicating the secretion of RFP (Figure 4a). Furthermore, *Z*‐stack confocal images of membrane‐stained fibroblasts that were allowed to interact with *E. coli+* for 18 h after IPTG induction revealed red fluorescence within their membranes, while those that interacted with *E. coli*− did not exhibit such fluorescence (Figure 4b). Due to the slow secretion of RFP, it is quite possible that very close membrane contact is necessary for RFP to cross the bacterial outer membrane and enter the cell through its cell membrane. In the absence of such close contacts, it is expected that RFP crossing the bacterial outer membrane simply diffuses into the culture medium at a very low concentration. This observation, even if it was unintended in our study, opens interesting possibilities for using bacterial biomaterials for light‐controlled targeted delivery of proteins from *E. coli* to mammalian cells.

**Figure 4:**
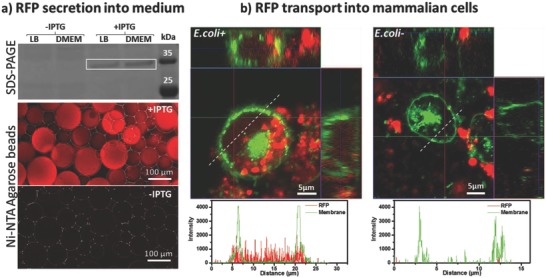
a) Analysis of RFP secretion into medium by SDS‐PAGE reveals RFP secretion (bands in white box) in *E. coli*+ culture after 16 h in the presence and absence of IPTG inducer. Experiments were performed in bacterial LB medium and in cell culture DMEM medium. Microscopy images (merged phase contrast and epifluorescence red channels) of Ni‐NTA agarose beads incubated with culture media of *E. coli+* grown in the presence and absence of IPTG indicates the presence of RFP in DMEM medium after 3 h. b) Laser scanning confocal *Z*‐stack orthogonal projection images of membrane‐stained fibroblasts interacting with *E. coli+* and *E. coli*−. Images correspond to fixed samples 18 h after IPTG induction. Red: RFP, Green: Cell membrane stain. The bright green area within the cells corresponds to the nuclear membrane. The plots below the microscopy images correspond to the intensity profile of red and green fluorescence along the dashed‐white line.

In summary, we have shown that a specially engineered endotoxin‐free *E. coli* strain can be integrated into a biomaterial and mediate specific interaction with mammalian cells, and this interaction can be temporally regulated by light. Bacterial surface display and optogenetic strategies have been combined to set up this system. We showed that bacterial surfaces can be easily prepared using PDL, bacterial growth can be controlled using tetracycline and light‐induced activation of surface immobilized bacteria can be achieved using PA‐IPTG. Specific adhesive interactions between integrins at the cell membrane and immobilized bacteria were observed through mRGD displayed on the bacterial surface. Due to the traction forces applied by cells, bacteria were pulled across the surface and accumulated around cells as they migrated. Finally, the unexpected observation that cells on *E. coli+* containing surfaces developed red fluorescence within their intracellular space provides an indication that this method can be used to deliver proteins from bacterial materials to cells.

Even though the light‐activatable chemical inducer, PA‐IPTG, used in this proof‐of‐concept study is an ideal tool to introduce light‐regulated protein expression in *E. coli* using commonly available vectors, it also imposes certain limitations on the system. Most *E. coli* strains express lactose‐permease in the presence of IPTG that actively transports the inducer within the cell. As a consequence, inhomogeneous protein induction occurs in a bacterial population with nonsaturating quantities of IPTG. This in‐turn limits the possibility of establishing precise dosing of protein expression by varying light‐activation intensities using PA‐IPTG.^25^ Furthermore, due to diffusion of the inducer, spatially confined activation cannot be easily achieved. However, the flexibility allowed by *E. coli* in terms of protein expression, together with the impressive evolution of tools in optogenetics allows light activatable protein induction systems to be genetically encoded directly within the bacterium.^33^, ^34^ In further studies, such systems will employed to elicit cellular responses to proteins that can be expressed in a precisely dosed, spatially confined, and possibly repeatable manner. Combined with the ability to incorporate activation at multiple visible wavelengths, such a system will open enormous possibilities for disruptive innovations in biomaterials science, within the emerging field of engineered living biomaterials.^35^

## Conflict of Interest

The authors declare no conflict of interest.

## Supporting information

Supplementary

Supplementary
