# Supplementary material for: Toward Light‐Regulated Living Biomaterials
Source: Adv Sci (Weinh). 2018 Jun 29;5(8):1800383. doi: 10.1002/advs.201800383 (PMC6097140; doi:10.1002/advs.201800383)
Supplement: Supplementary file 1 — Supplementary [file ADVS-5-1800383-s002.pdf]

Copyright WILEY-VCH Verlag GmbH & Co. KGaA, 69469 Weinheim, Germany, 2018.

ADVANCED SCIENCE
Open Access

Supporting Information

for *Adv. Sci.,* DOI: 10.1002/advs.201800383

Toward Light-Regulated Living Biomaterials

Shrikrishnan Sankaran, Shifang Zhao, Christina Muth, Julieta Paez, and Aránzazu del Campo**

Copyright WILEY-VCH Verlag GmbH & Co. KGaA, 69469 Weinheim, Germany, 2016.

Supporting Information

Towards Light-Regulated Living Biomaterials

Shrikrishnan Sankaran, * *Shifang Zhao, Christina Muth, Julieta Paez, Aránzazu del Campo* *

Dr. S. Sankaran, S. Zhao, C. Muth, Dr. J. Paez, Prof. Dr. A. del Campo
Dynamic Biomaterials, INM – Leibniz Institute for New Materials, Campus D2 2, 66123 Saarbrücken, Germany
Email: shrikrishnan.sankaran@leibniz-inm.de, aranzazu.delcampo@leibniz-inm.de

Prof. Dr. A. del Campo, S. Zhao
Chemistry Department, Saarland University, 66123 Saarbrücken, Germany

Materials and Methods

N-hydroxysuccinimide (NHS) functionalized Nexterion coverslip H (Schott, Material code: 1098523), silicon gasket (ibidi GmbH, Material code: 81201), Poly-D-lysine (SERVA Electrophoresis GmbH), were purchase from specified companies. Sterile Dubelcco's Phophate-Buffered Saline (PBS) was purchased from ThermoFisher Scientific. All water used in the work was ultrapure water with a resistivity of 18 MΩ.cm.

6-nitro piperonal was purchased from Alfa Aesar (Karlsruhe, Germany). All other reagents were obtained from Sigma-Aldrich (Steinheim, Germany) unless otherwise specified.

Analytic/ preparative RP-HPLC was performed on a Jasco MD 4000 system equipped with a diode-array detector, a fraction collector, and Reprosil-100, C18 (5µm) columns. Elution was performed using a gradient of 0.1%TFA in water to 95%ACN+ 5% H2O +0.1% TFA.

Solution 1H-NMR-NMR spectra were recorded at 25 °C on a Bruker Advance III, 300MHz, (Karlsruhe, Germany). All measurements were taken at room temperature, by employing tetramethylsilane (TMS) (δ= 0 ppm) as internal reference. The chemical shifts are given in parts per million. The following abbreviations are used: s-singlet, t-triplet, q-quartet, m-multiplet. Electrospray ionization mass spectra (ESI-MS) were recorded with a LC-ESI (Agilent, Germany) in the positive detection mode.

Microscopy images and time-lapse recordings were acquired with 20x and 63x objectives using either a Cell Observer inverted microscope (Zeiss Axio Observer Z1) controlled by ZEN blue software, a confocal microscope (Zeiss LSM 880) controlled by ZEN black software or an inverted Nikon Ti-Eclipse microscope controlled by NIS Elements software. All microscopes were equipped with an incubation chamber which maintained the temperature at 37°C and CO_2 concentration at 5%.

Image processing and analyses were performed using Fiji edition of ImageJ.[1]

Synthesis of photoprotected IPTG ((4aR,6S,7R,8R,8aR)-6-(Isopropylthio)-2-(6-nitrobenzo[d][1,3]dioxol- 5-yl)hexahydropyrano[3,2-d][1,3]dioxine-7,8-diol):

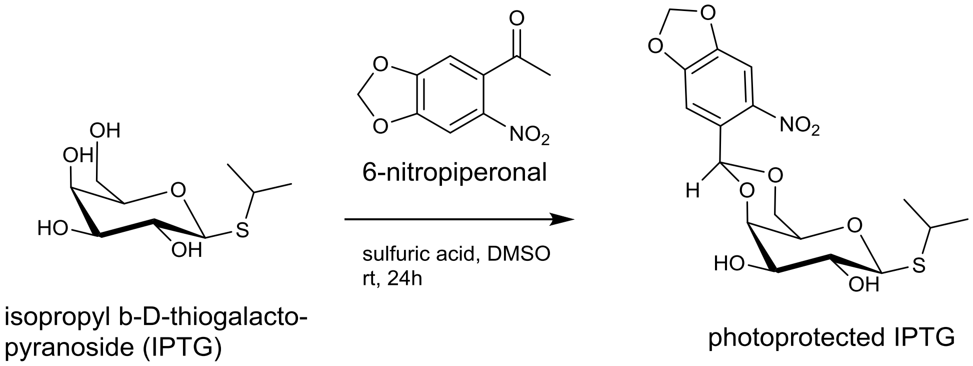

A reported protocol[2] was followed with some modifications. Isopropyl-b-D-thiogalactoside (IPTG, 43 mg, 0.18 mmol, 1eq) was dissolved in DMSO (1 mL) and cooled to 0°C in ice bath. 6-Nitropiperonal (37 mg, 0.18 mmol, 1 eq) was added, followed by concentrated sulfuric acid (0.4 mL). The mixture was allowed to reach room temperature, and then stirred for 24h. Subsequently, the reaction was quenched with water and extracted with ethyl acetate three times. The combined organic layers were washed with water and brine, dried over magnesium sulfate and evaporated. The residue was purified by RP-HPLC (20B to 95B, detection at 360 nm), evaporated and freeze dried. A pale-yellow solid was obtained (Y= 10% in our hands). Characterization matched the reported data in the literature. 1H NMR (300 MHz; CDCl3): δ (ppm)= 7.44 (s, 1H), 7.35 (s, 1H), 6.19 (s, 1H), 6.12 (s, 2H), 4.40 (m, 1H), 4.30 (m, 2H), 4.07 (m, 1H), 3.70 (m, 2H), 3.52 (m, 1H), 3.25 (m, 1H), 1.36 (m, 6H). ESI-MS+: 432.8 (M+NH4)+, 437.6 (M+Na)+.

Construction of plasmids and bacterial strains

pETDuet-1 vector was purchased from Merckmillipore. pRsetB-TagRFP was a kind gift from Dr. Dorothee Wasserberg from the University of Twente. The TagRFP gene was amplified with NdeI and XhoI restriction sites with which it was inserted into the second MCS of pETDuet-1.

The eCPX gene was taken from plasmid pB33eCPX, which was a gift from Patrick Daugherty (Addgene plasmid # 23336). The mRGD gene was purchased as a synthesized oligonucleotide by Eurofins MWG Operon. BsrGI and NheI restriction sites were introduced between the OmpXss and eCPX gene in the pB33eCPX plasmid using the Q5 site directed mutagenesis kit from NEB. mRGD was amplified by PCR with the BsrGI and NheI restriction sites, which were then used to clone it between OmpXss and eCPX in pB33eCPX. OmpXss-mRGD-eCPX was then amplified with SacI and HindII sites with which they were cloned into the first MCS of pETDuet-1 to yield the plasmid named pD-mRGD-eCPX-RFP.

The scrambled mRDG version was constructed by site directed mutagenesis of the above-mentioned plasmid using the Q5 site directed mutagenesis kit by NEB to yield pD-mRDG-eCPX-RFP. Similarly, His6 tag was added to the C-terminal of eCPX using site directed mutagenesis to yield pD-mRGD-eCPX-H6-RFP.

All plasmids were transformed by electroporation into ClearColi Bl21(DE3) cells from BioCat exactly as specified by the provider. Bio-Rad Micropulser™ Electroporator was used in combination with Bio-Rad 0.1 cm electroporation cuvettes (1652083).

pETDuet1 – mRGD-eCPX, RFP Vector Map

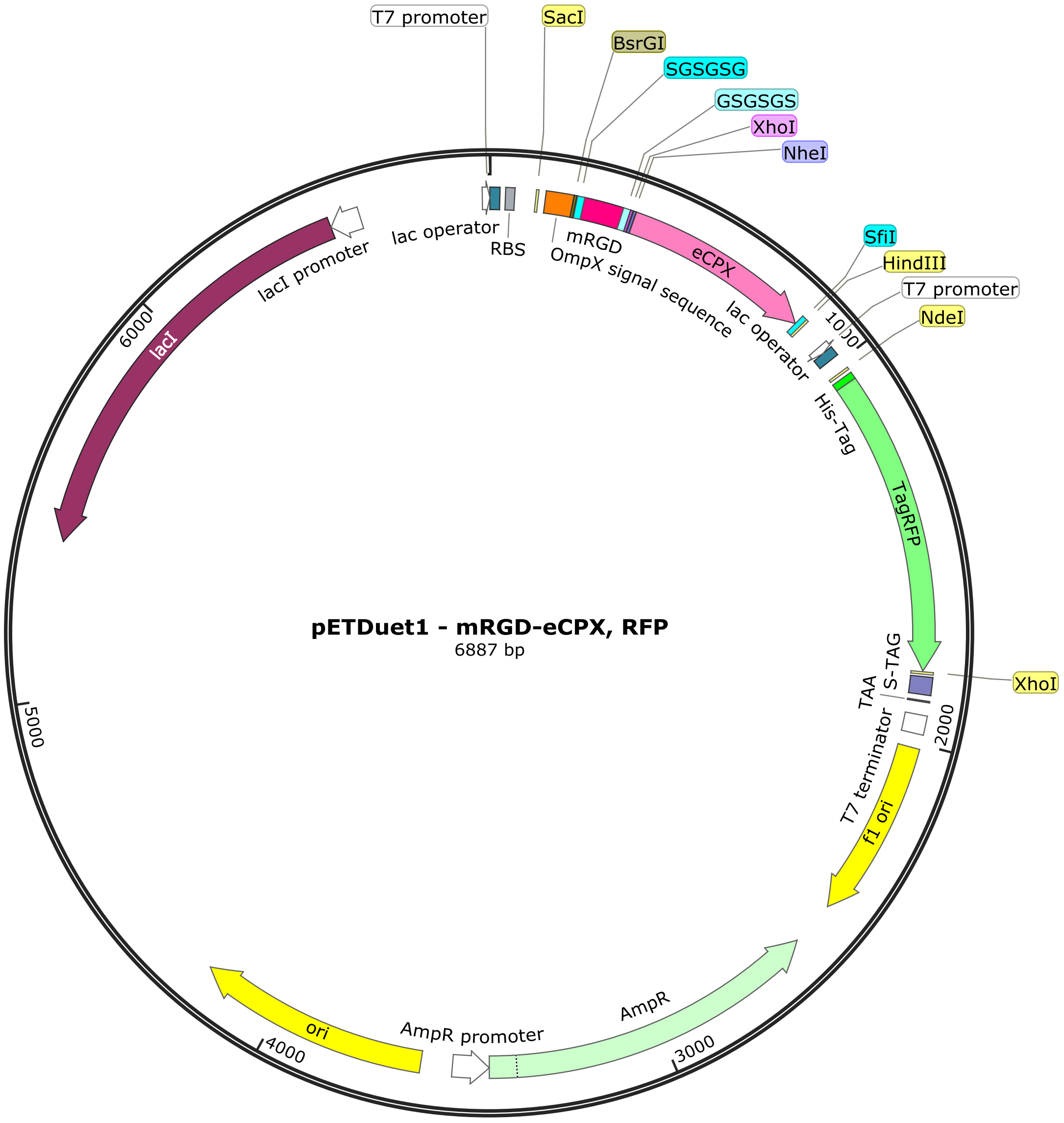

Gene sequences

OmpX signal sequence:

atgaaaaaaattgcatgtctttcagcactggccgcagttctggctttcaccgcaggtacttccgtagct

mRGD:

ggctgcccgcgcccgcgcggcgataacccgccgctgacctgcagccaggatagcgattgcctggcgggctgcgtgtgcggcccga
acggcttttgcggc

eCPX:

ggagggcagtctgggcagtctggtgactacaacaaaaaccagtactacggcatcactgctggtccggcttaccgcattaacgactggg
caagcatctacggtgtagtgggtgtgggttatggtaaattccagaccactgaatacccgacctacaaacacgacaccagcgactacggt
ttctcctacggtgcgggtctgcagttcaacccgatggaaaacgttgctctggacttctcttacgagcagagccgtattcgtagcgttgacg

taggcacctggattttgtctgttggttaccgcttcgggagtaaatcgcgtcgcgcgacttctactgtaactggcggttacgcacagagcgacgctcagggccaaatgaacaaaatgggcggtttcaacctgaaataccgctatgaagaagacaacagcccgctgggtgtgatcggttcttcacttacaccgagaaaagccgtactgcaagctaataa

TagRFP:

atgagcgagctgattaaggagaacatgcacatgaagctatacatggagggcaccgtgaacaaccaccacttcaagtgcacatccgagggcgaaggcaagccctacgagggcacccagaccatgagaatcaaggtggtcgagggcggccctctcccctcgccttcgacatcctggctaccagcttcatgtacggcagcagaaccttcatcaaccacacccagggcatccccgacttctttaagcagtccttccctgagggcttcacatgggagagagtcaccacatacgaagacgggggcgtgctgaccgctacccaggacaccagcctccaggacggctgcctcatctacaacgtcaagatcagaggggtgaacttcccatccaacggccctgtgatgcagaagaaaacactcggctgggaggccaacaccgagatgctgtaccccgctgacggcggcctggaaggcagaagcgacatggccctgaagctcgtgggcgggggccacctgatctgcaacttcaagaccacatacagatccaagaaacccgctaagaacctcaagatgcccggcgtctactatgtggaccacagactggaaagaatcaaggaggccgacaaagagacctacgtcgagcagcacgaggtggctgtggccagatactgcgacctccctagcaaactggggcacaagg

mRDG:

ggctgcgtgaccggccgcgatggcagcccggcgagcagctgcagccaggatagcgattgcctggcgggctgcgtgtgcggcccgaacggcttttgcggc

His6-Tag:

catcatcatcaccatcac

Outer membrane and cytosolic proteins sub-fractionation

The bacterial cultures were grown in 50 ml LB Miller medium with 50 µg/ml Ampicillin (Roth #HP62) at 37°C, 250 rpm and appropriate cultures were induced at 0.5-0.8 OD with 0.1mM IPTG. The cultures were then harvested as spun-down pellets after 4h of growth (3300 x g, 20 min, 4°C, Hettich Rotanta 460 RS), quick-frozen in liquid Nitrogen and stored at -20°C.

The sub-fractionation was performed using a modified version of a previously reported strategy by Thein and coworkers (Method 1).[3] In short, pelleted cells were resuspended in 1 ml 0.2 M Tris-HCl pH 8, 1 Mbsucrose, 1 mM EDTA. 100 µL of lysozyme (Roth) (5 mg/mL in MQ) were added, vortexed and incubated for 5 min at RT. Four ml of MQ were added and incubated for 20 min at RT until spheroblast formation was observed under the microscope. Then 6 mL 50 mM Tris-HCl pH 8, 2% (w/v) Triton X-100, 10 mM MgCl2 and 12 µL Benzonase Nuclease (Novagen Merck, 300 U) were added and mixed until the suspension was clear. The mixture was centrifuged at 75 000× g, 60 min, 4 °C (Beckman Avanti J26XP JA 25.50 Rotor 10 ml PC vials).

The pellets containing the outer membrane fraction were resuspended in 250 µL 50 mM Tris-HCl pH 8, 2% (w/v) Triton X-100, 10 mM MgCl2.

The supernatants were concentrated 4x using 3 kDa Pal Microsep centrifugal devices at 3300 g, 2.5 h, 4°C, Hettich Rotanta 460 RS.

SDS-PAGE and Western Blot

The *E. coli* strain carrying the plasmid pD-mRGD-eCPX-H6-RFP that results in the expression of a His6-tagged variant of mRGD-eCPX, named mRGD-eCPX-His6, was used in the following experiment. 20 µL sample (pellets and conc. supernatants) and 7 µL Laemmli 4x buffer were mixed and heated at 95°C for 5 min. 10 µl of each was loaded on 12% SDS gels and run at 120 V for 60 min. One gel was Biosafe Coomassie (Bio-Rad) stained, the second one was wet blotted to a PVDF membrane (Merck Millipore) with Mini Trans–Blot

Cell (Bio-Rad) at 100V for 1h. Fluorescent antibody staining was performed with SNAP i.d. 2.0 (Millipore). As primary antibody Anti-His-TAG from mouse (Invitrogen/FisherScientific #10755503) was used and the secondary antibody was an Anti-mouse IgG(H+L), Cy 3 conjugated from goat (Dianova #115-165-146) both diluted 1:500 in PBST. Pictures were taken in RGB multiplex mode of Fluorchem Q imager (ProteinSimple).

Bacterial surface preparation and Cell culture

The Nexterion coverslip was divided in 12 wells (0.56 cm^2 per well) by placing a silicone gasket on the top. 50 µL Poly-D-lysine solutions (2 mg/ml in PBS) were incubated in the wells for 60 min, allowing covalent immobilization of the molecules by reaction of their amine groups with the activated carboxylic acids at the Nexterion surface. Substrates were blocked by immersing in 50mM ethanolamine in PBS for 60 min and rinsed with water 3 times. Before the cell experiment, the substrates were sterilized by incubating in 70% ethanol for 5 min and rinsed with sterile PBS 3 times.

To immobilize bacteria on these surfaces, bacteria were inoculated from glycerol stocks in 5 mL LB broth containing 50 µg/mL ampicillin and grown overnight at 30°C, 250 rpm. The bacterial densities typically reached ~0.5 O.D.$_{600nm}$ by the morning. The cultures were then spun down at 3500 rcf for 10 mins and the pellets were resuspended in in sterile PBS with O.D.$_{600nm}$ 1.0. 100 µL of these bacterial solutions were incubated for 30 min in each well. These surfaces were then washed 3 times with sterile PBS by vigorous pipetting.

WT eGFP-Vinculin mouse embryonic fibroblast lines (MEF-vincGFP) were cultured in DMEM with 10% fetal bovine serum, 1% Nonessential Amino Acids, 1% Sodium Pyruvate and 4 mM L-Glutamine (all of them purchase from GIBCO) in a humidified incubator at 37°C/5%CO_2. For every experiment, the cells were trypsinized and the cell density was determined a TC20™ Automated Cell Counter (Bio-Rad). The cells were directly seeded in the wells at a density of 2.5 x 10^4 cells / well. For all experiments with bacteria, the cell-culture medium was supplemented with 50 µg/mL ampicillin.

Bacterial growth control assay

The *E. coli+* bacterial surfaces were incubated in the DMEM-based cell culture medium containing 50 µg/mL ampicillin. Appropriate amounts of tetracycline and 0.1 mM IPTG were added when necessary as mentioned. Live-cell imaging was performed using the Nikon Ti-Eclipse microscope with a 20x objective. Images were taken every 20 min for 22 h in both phase contrast and red fluorescence channels. After 22 h, all samples were gently washed 3 times with PBS and DMEM-based cell culture medium along with appropriate additions were added. Live-cell imaging was then repeated for another 7 h. Bacterial surface density analysis was performed by quantifying the area of the background, where bacterial cells had not grown. Fluorescence intensity values were determined by quantifying the mean-grey values of the fluorescing bacteria in the red channel minus the background value.

Induction of protein expression

Three strategies were used to induce protein expression in the surface-immobilized bacteria using PA-IPTG and IPTG. PA-IPTG stock solution was prepared in DMSO at a concentration of 500 mM and IPTG stock solution was prepared in water at a concentration of 100 mM.

(i) PA-IPTG was diluted in the appropriate medium at a final concentration of 500 nM and this solution was exposed to 360 nm irradiation using a Lumos 43 illuminator (Atlas photonics) for 1 min. This medium was then added on the bacterial surfaces.
(ii) PA-IPTG was diluted in the appropriate medium at a final concentration of 500 nM and added on the bacterial surfaces in the dark. When required, 360 nm light was

irradiated from the Zeiss Axio Observer Z1 microscope using a Colibri 2/365 nm LED module light source at 50% intensity and an EC-Plan NeoFluar 20x objective for 2 min. When only bacteria were present, the objective was scanned over the whole well and when MEF-vincGFP cells were also present, irradiation was done only in one corner of the well.

(iii) IPTG was added to the medium at a final concentration of 100 nM

Sample fixation and staining

For high magnification and confocal imaging, samples were fixed by washing 3 times with sterile PBS then incubating for 15 min with 4% paraformaldehyde (PFA) solution in PBS then washing again 3 times with sterile PBS. The cell nuclei were stained using DAPI and mounted using standard protocols.

Sample Preparation for Scanning Electron Microscopy and Image acquisition

The samples were washed with PBS, fixed for 30 min with 2 wt% glutaraldehyde in 0.1 M Cacodylat (214 g/mol), and then washed 3 times with sterile PBS. In order to dehydrate, the samples were immersed into ethanol with increasing concentrations, 30% for 10 min, 50% for 10 min, 70% for 10 min, 80% for 10 min, 90% for 10 min, 96% for 10 min, 100% for 2x15 min. Subsequently, the sample was incubated in 100% ethanol mixed at a 1:1 volume ratio with hexamethyldisilazan (HMDS) for 15 min. After that, samples were immersed into pure HMDS for 15 min twice. At the end, the samples were dried in an exhaust fume hood. The surface of the cell membrane was observed using the scanning electron microscope (SEM) (JSM-7500F; JEOL).

RFP Secretion test

5 mL bacterial cultures were grown from glycerol stocks to an $O.D._{600nm}$ of ~0.5 as already described. The cultures were split in 2 parts then spun down and resuspended either in LB medium or DMEM-based cell culture medium with 50 µg/mL ampicillin. These cultures were further each split in 2 parts. In one part 0.1 mM IPTG was added and in the other nothing was added. The cultures were incubated at 37°C 250 rpm. After 3 h of incubation, 100 µL of the cultures were taken and spun down at 6000 rcf for 5 min and 50 µL of the medium was pipetted out from the top and incubated with 10 µL of Ni-NTA agarose beads (Qiagen) for 5 min. The beads were then spun down at 1000 rcf for 30s and washed 2 times with PBS. The bead solutions were placed in 96 well-plate wells and imaged using the Nikon Ti-Eclipse microscope. After 16h of incubation, the cultures were spun down and 20 µL of the medium was pipetted out from the top and used for SDS-PAGE analysis, performed as already described.

Sample Preparation for Membrane Labeling and Image acquisition

L929 fibroblast cells were seeded on *E. coli+* or *E. coli-* bacterial surfaces and allowed to interact for 18 h in the presence of 100mM IPTG and 2 µg/mL of Tetracycline. After that, the samples were washed with Hank’s balanced salt solution (HBSS). Wheat germ agglutinin conjugates Oregon Green® 488 (WGA, invitrogen) was diluted to 5 µg /ml by HBSS to get the working concentration. This labeling solution was added to cover cells and was incubated for 10 minutes at 37°C. When labeling was complete, the labeling solution was removed, and cells were washed twice with 200 µL HBSS. Then samples were mounted in pre-warmed HBSS buffer for imaging.

The fluorescence of the L929 cell membrane and RFP were observed using a confocal microscope (Zeiss LSM 880) controlled by ZEN black software. For the quantification and comparison of the fluorescence intensity, all the Z-stacks images were acquired under the

same conditions. To assess the fluorescence intensity, a line scan cross the cell membrane was performed and the plot profile of fluorescence was measured. The xy, xz and yz optical sections of Z-stacks were generated by orthogonal view of ZEN black software.

Supporting information Figures

Control of bacterial growth using tetracycline

Addition of tetracycline at concentrations spanning 1 – 10 µg/mL slowed down bacterial growth, with nearly no growth at 10 µg/mL (Figure 1b). When tetracycline was removed and IPTG added, the bacterial surfaces developed red fluorescence at similar rates indicating that no permanent damage was imparted to the bacterial cells' ability to produce heterologous proteins (Figure 1c). Bacterial surfaces exposed to 10 and 5 µg/mL of tetracycline showed a slightly faster rate of production and higher intensity of red fluorescence compared to the other surfaces, most likely since the bacteria did not overgrow into a possible overcrowded saturation phase. In all cases, red fluorescence reached detectable levels after approximately 90 mins, fitting well with previously reported half-time for the maturation of the protein.[36] In the presence of both tetracycline and IPTG, it was seen that protein expression slowed down progressively with increasing tetracycline concentrations (Figure 1e). Notably, protein expression, induced by IPTG, also delayed bacterial growth even in the absence of tetracycline and a lower tetracycline concentration was required to arrest the growth over several hours (Figure 1d). Hence in further experiments, 10 µg/mL tetracycline was used before induction of gene expression and either 2 µg/mL or 0 µg/mL was used after induction when the duration of the experiment was longer or shorter than 10 h respectively.

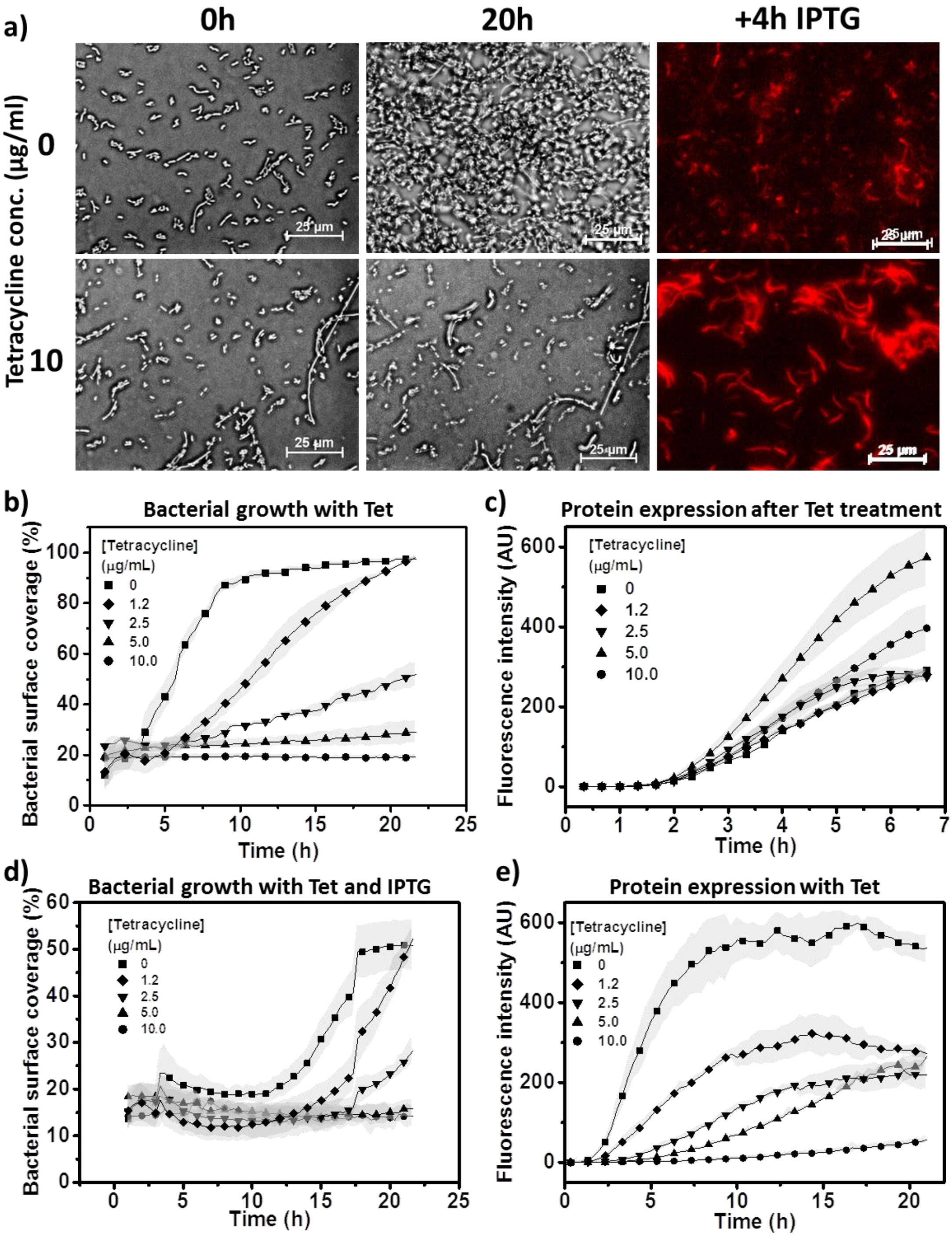

Figure S1. a) Phase contrast images of bacterial cells growing on Nexterion® glass slides coated with PDL in the presence and absence of tetracycline at 0 and 20 h timepoints. The right panels represent IPTG induced expression of RFP after the tetracycline treatment. b) Surface coverage of bacterial cells in the presence of different tetracycline concentrations over time. c) Development of red fluorescence due to IPTG induce protein expression in the surface-confined bacterial cells after the tetracycline treatment. d) Surface coverage of bacterial cells in the presence of 0.1 mM IPTG and different concentrations of tetracycline. e) IPTG-induced expression of RFP quantified over time in surface-immobilized bacterial cells in the presence of different concentrations of tetracycline. The grey bands in all plots represent standard deviation obtained from 3 individual samples in each case.

Light activation of PA-IPTG

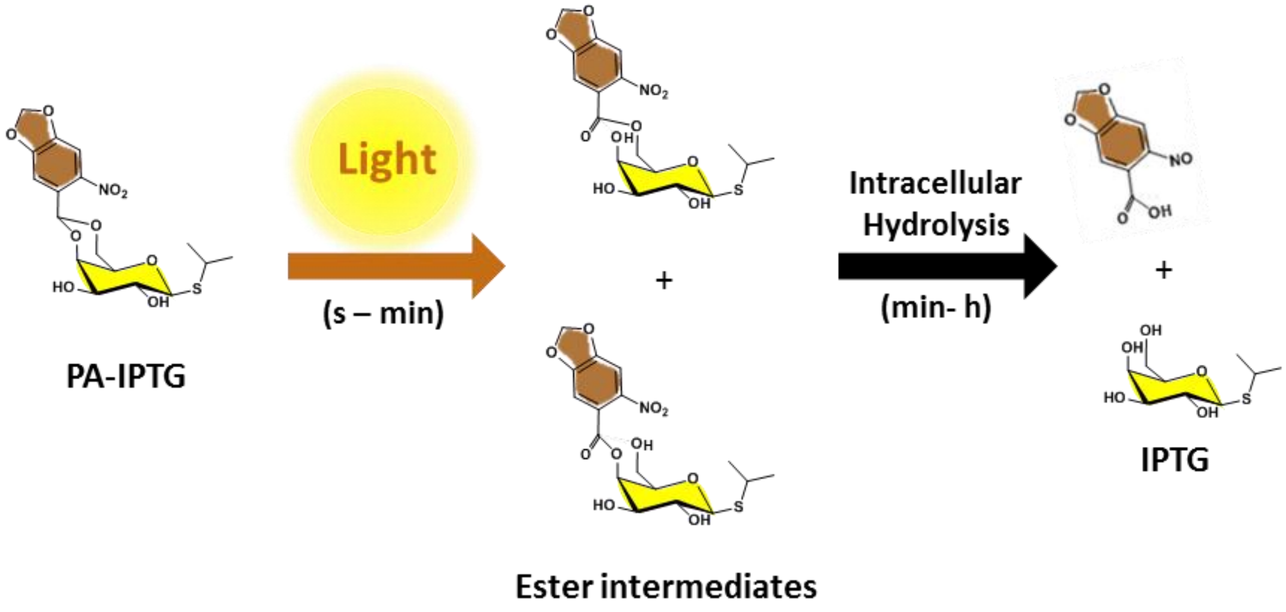

Figure S2. Two-step light-responsive activation mechanism of PA-IPTG. On irradiation with 360 nm light, PA-IPTG rapidly forms regioisomeric NP-nitrosocarbonyl esters as intermediates that then get hydrolyzed to active IPTG by intracellular esterases.

Mammalian cell response to IPTG-induced bacterial surfaces

MEF-vincGFP cells were seeded on bacterial surfaces after which 0.1 mM IPTG was added into the medium to induce bacterial protein expression. The cells recognized and rapidly pulled the *E. coli*+ bacteria off the surface. This seemed to happen in all directions around the MEF-vincGFP cells and resulted in the cells migrating over the surface and accumulating *E. coli*+ cells under them (Figure 2a). As expected, the cells showed on interaction with the activated *E. coli*- cells (Figure 2b). Z-stack images of MEF-vincGFP cells on *E. coli*+ surfaces, obtained using laser-scanning confocal microscopy, show focal adhesions completely enveloping the bacteria, indicating that the cells used some sort of gripping mechanism to pull the bacteria off the surface (Figure 2c), while no such interaction was seen with *E. coli*- (Figure 2d). SEM images further revealed cell protrusions completely engulfing the bacteria (Figure 2e,f). The bacteria were seen both below and above the cell extensions and some even emerging from underneath through pores.

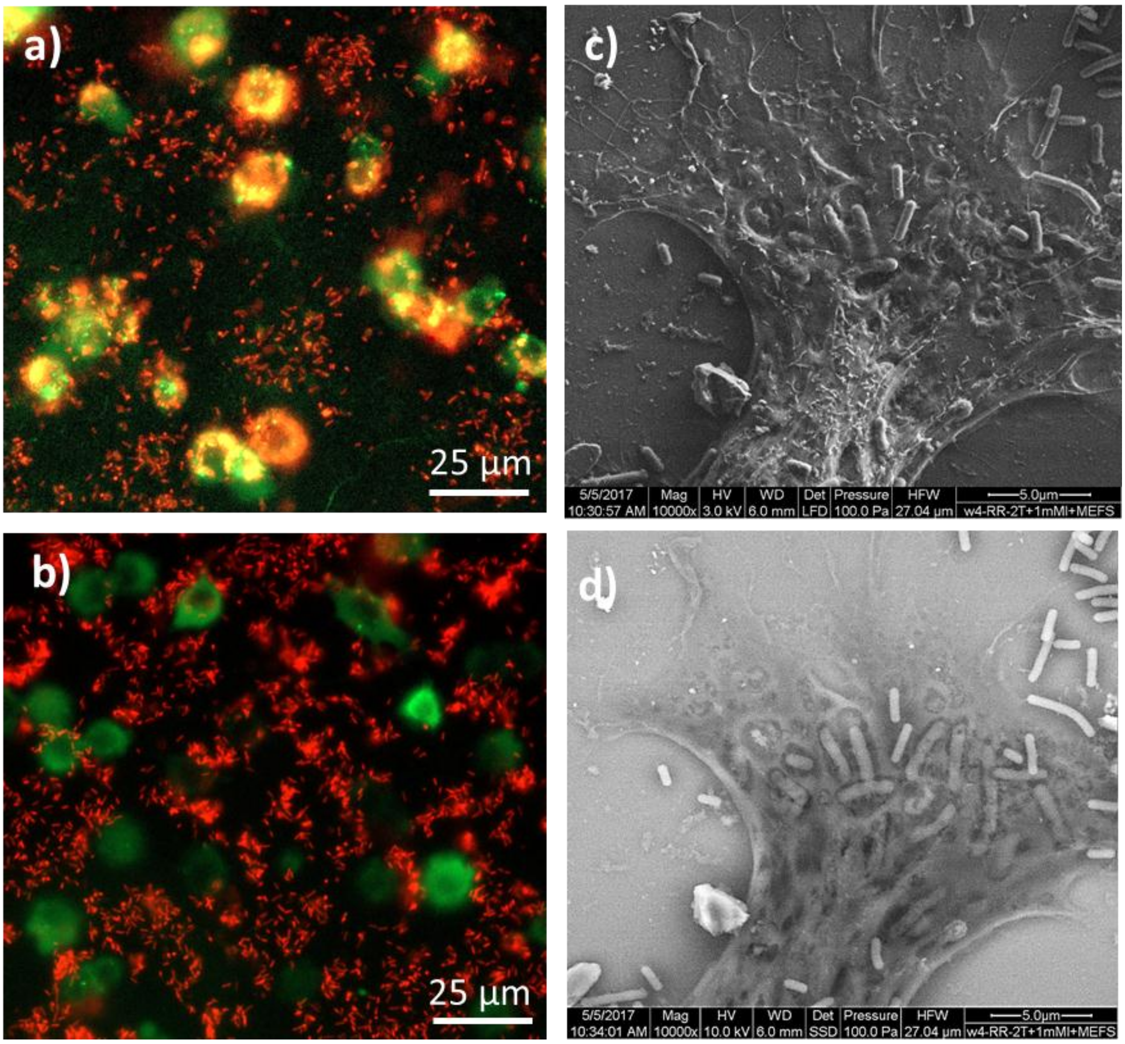

Figure S3. Epifluorescence images of MEF-vincGFP cells colocalized with bacterial cells in the case of a) *E. coli+* and completely separated in the case of b) *E. coli-*. Red: bacteria, Green: vincGFP in MEF-vincGFP cells. SEM images of a cellular protrusion interacting with *E. coli+* obtained using c) Secondary electron and d) backscattered electron detection methods. All images are of samples fixed 10 h after IPTG induction was performed.

SEM images of mammalian cells interacting with light-activated *E. coli*

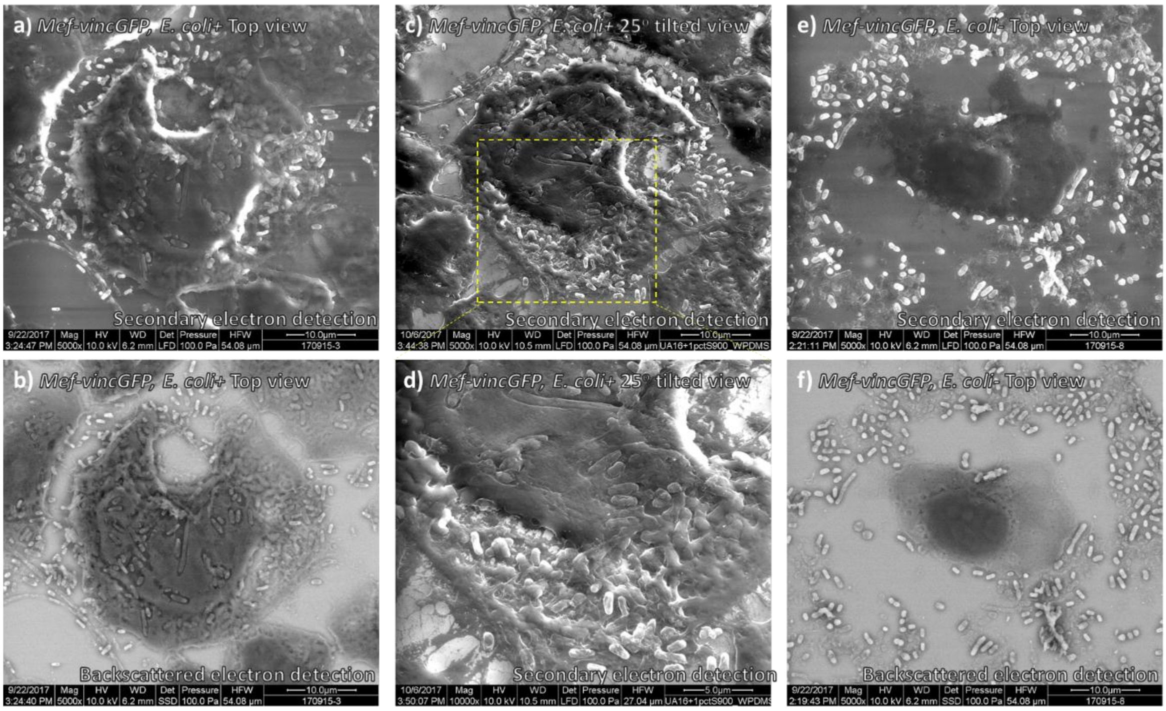

Figure S4. SEM images of MEF-vincGFP with *E. coli+* obtained by a) secondary electron detection and b) backscattered electron detection. c) The same cell was imaged at an angle of 25° and d) with higher magnification showing bacterial cells above and below a cell protrusion. MEF-vincGFP with *E. coli-* obtained by e) secondary electron detection and f) backscattered electron detection.

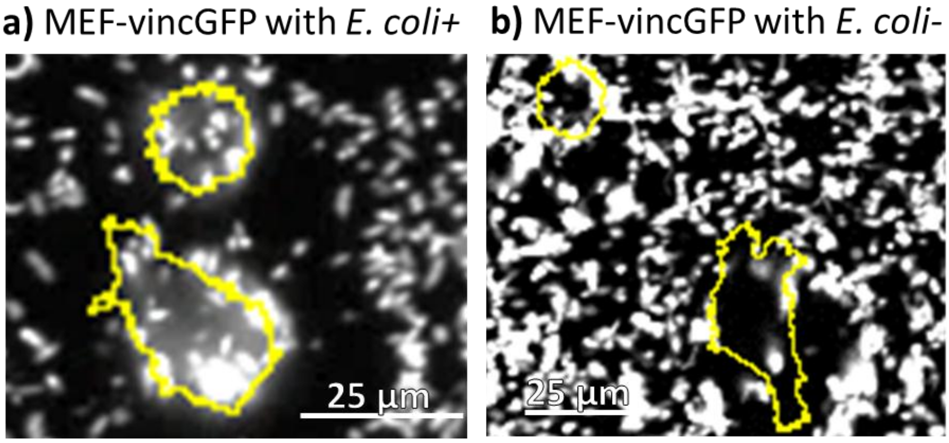

Figure S5. Fluorescent haze visible within the boundaries of MEF-vincGFP cells (approximate outlines in yellow) in the case of a) *E. coli+* and not visible with b) *E. coli-* cells.

Supporting information videos:

SI Video S1: Phase-contrast and Red fluorescence merged channel time-lapse imaging of *in situ* light-activated *E. coli+* in the presence of MEF-vincGFP cells. Time counter format is in hh:mm and starts 1h after light-activation.

References:

[1] J. Schindelin, I. Arganda-Carreras, E. Frise, V. Kaynig, M. Longair, T. Pietzsch, S. Preibisch, C. Rueden, S. Saalfeld, B. Schmid, J.-Y. Tinevez, D. J. White, V. Hartenstein, K. Eliceiri, P. Tomancak, A. Cardona, *Nat. Methods* **2012**, *9*, 676.
[2] D. D. Young, A. Deiters, *Angew. Chem. Int. Ed.* **2007**, *46*, 4290.
[3] M. Thein, G. Sauer, N. Paramasivam, I. Grin, D. Linke, *J. Proteome Res.* **2010**, *9*, 6135.
